# 

**Published:** 1916-04-22

**Authors:** 


### CONTENTS.

PAGE

**The Enrolment Scheme and the Profession** ... 73

**Medical Fees Within the Profession** ... .. 74

**Hospital and Institutional News** ... ... ... 75

The Late B. Burford Rawlings—The Inspector of Military Orthopædics—The Military Heart Hospital at Hampstead—A Case of Leprosy in London—State-Aided Treatment of Venereal Diseases—M.P.s' Salaries for Hospitals—Taxation of Doctors' Cars—A Gift of Land to the Royal Free Hospital—" Named Beds " Productiveness in Scotland—The Scottish Hospital for Limbless Warriors : £52,000 in a Month—The Saturday Fund's Success—The Southern Hospital, Liverpool, and a Local Wager—Medicinal Herb Cultivation in Hospital Gardens—School Inspection in Leeds—X-Ray Assistants—Some Massage Howlers—Where to Treat Our Colonial Wounded—This Week's Drug Market.

**The Problem of Tuberculosis** ... ... ... 79

The Patient, the Nurse, and Questions of Economy.

**The Human Body** ... ... ... ... ... ... 80

Its Plan of Organisation.

**A Medical Causerie** ... ... ... ... ... ... 83

**Parliament and the Profession** ... ... ... 84

**Child Welfare Work in Bradford** ... ... ... 85

Hospital and Municipal Work Compared.

**University Education in Wales** ... ... ... 87

A Royal Commission Appointed.

PAGE

**Legal Notes for Institutional Workers** ... ... 86

Medical Officers and Old-Age Pensioners—Bequests to Institutions—Workmen's Compensation and *Post-Mortems*.

**" National Egg Scandal "** ... ... ... ... 87

**The Hospital Laundry** ... ... ... ... ... 88

I.—Its Constituent Parts, Equipment, and Staff.

**St. George's Hospital** ... ... ... ... ... 88

**The Matrons' and Sisters' Department** ... 89

EASTER DAY, 1916.

Round the Hospitals : Miss Nutting on Nursing Conditions—The Trolley Women Workers—The R.B.N.A. and the College—An Idea for Out-Patients—"Wounded in the dardanelles "—Tripe Diet.

**The Editor's Letter-Box** ... ... ... ... 91

A Matron's Worries—A Correction—Ambulance Dogs—Some Difficulties with V.A.D. Workers.

**Matrons' and Sisters' Appointments** ... ... 92

**Hospital Results in 1915** ... ... ... ... 92

More Early Reports of the Voluntary Hospitals.

**Passing Events and Topics** ... ... ... ... iv

**Gifts to Hospitals** ... ... ... ... ... iv

**The Book Market** ... ... ... ... ... iv

**The Institutional Worker** ... ... (Suppl.) 1

The Depletion of Male Hospital Staffs : Answer to March Question (No. 1).

Design for a Wardrobe.

